# Analysis of the clinical factors affecting the negative rate of metagenomic next-generation sequencing in patients with spinal infection

**DOI:** 10.1097/JS9.0000000000001913

**Published:** 2024-07-11

**Authors:** Guang Zhang, Qianfei Liu, Dan He, Xiaojiang Hu, Yanbing Li, Mingxing Tang, Shaohua Liu, Weiqiong Liu, Hongqi Zhang, Qile Gao

**Affiliations:** a Department of Spine Surgery and Orthopaedics, Xiangya Hospital, Central South University; b National Clinical Research Center for Geriatric Disorders, Xiangya Hospital, Central South University; c Department of Neurology, The First Hospital of Changsha; d Department of Clinical Laboratory, Xiangya Hospital, Central South University, Changsha, People’s Republic of China; e Hunan Sagene Medical Laboratory Co. Ltd, Changsha, China

Highlights

- Metagenomic next-generation sequencing (mNGS) had high sensitivity and specificity in the diagnosis of spinal infections.
- mNGS is far superior to traditional detection methods for pathogens of spinal infections.
- The positive rate of mNGS results from samples was significantly higher for those obtained by debridement than by needle biopsy, and the rate for pus and soft tissue was significantly higher than that for vertebral bone tissue.

*Dear Editor,*

We found a fascinating clinical phenomenon: mNGS has been widely used in diagnosing infectious diseases, showing favorable diagnostic performance. However, it was found that a high percentage of patients with spinal infectious had negative mNGS results. Meanwhile, the etiology of spinal infection is complex, and if untreated, spinal infection has serious consequences^1,2^. The key to its treatment is the rapid and accurate identification of pathogenic microorganisms and targeted anti-infective therapy^3^. The diagnosis of spinal infection is primarily based on pathological examination and pathogenic examination of needle biopsy or debridement surgery sampling^4,5^, but the positive rate of bacterial culture is not high, and pathological examination lacks specificity^6^. Therefore, we conducted a multicenter retrospective study to explore the clinical factors underlying the negative mNGS rate in patients with spinal infections.

Clinical data of 340 patients with spinal infections from four tertiary hospitals in China between January 2019 and March 2024 were included in the study. All patients underwent puncture biopsy or debridement surgery, and specimens were sent for mNGS, histopathology, and microbial culture. Case inclusion criteria: diagnosed as infectious lesions by histopathology and pathogenesis; and effective diagnostic anti-infective treatment. Patients without mNGS testing or with a clinical diagnosis of nonspinal infection were excluded.

A total of 340 patients with spinal infections were included in the study. Among the 340 samples, 282 were mNGS positive (245 from debridement surgery, 37 from needle biopsy), while 58 were mNGS negative (30 from debridement surgery, 28 from needle biopsy). The negative rate for mNGS was significantly lower in debridement surgery samples (10.91%) compared to needle biopsy samples (43.08%) (*P*<0.001) (Table 1, Fig. 1A). Similarly, soft tissue (13.91%) and pus samples (8.96%) had lower negative rates than vertebral tissue samples (31.65%) (*P*<0.001); There was no significant difference between soft tissue and pus samples (*P*>0.05) (Table 1, Fig. 1B).

**Table 1:** The basic characteristics of metagenomic next-generation sequencing negative group and positive group were analyzed.

Variables	Total (*n*=340)	mNGS+ (*n*=282)	mNGS− (*n*=58)	*P*
Age, median (Q1, Q3)	59 (51, 68)	59 (51, 68)	57.5 (52, 69)	0.711
Sex, *n* (%)				0.095
Female	148 (44)	129 (46)	19 (33)	
Male	192 (56)	153 (54)	39 (67)	
Duration, *n* (%)				1
＜3mouth	159 (47)	132 (47)	27 (47)	
≥3mouth	181 (53)	150 (53)	31 (53)	
Premedicate, *n* (%)				0.076
No	172 (51)	136 (48)	36 (62)	
Yes	168 (49)	146 (52)	22 (38)	
Biopsy, *n* (%)				<0.001
Needle	65 (19)	37 (13)	28 (48)	
Surgery	275 (81)	245 (87)	30 (52)	
Sample, *n* (%)				<0.001
Pus	67 (20)	61 (22)	6 (10)	
Soft tissue	194 (57)	167 (59)	27 (47)	
Vertebral body	79 (23)	54 (19)	25 (43)	

mNGS+/−, positive and negative metagenomic next-generation sequencing results.

**Figure 1:**
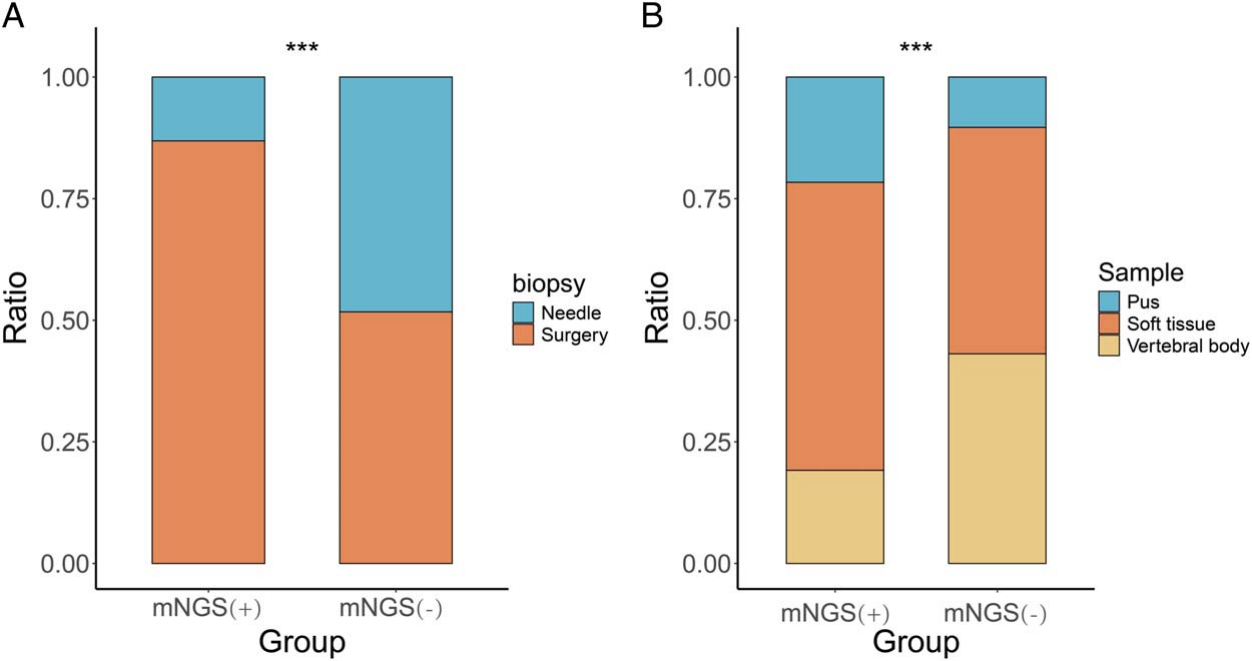
mNGS detection results of different sampling methods and different sample types. (A) The proportion of puncture biopsy and debridement in the positive and negative mNGS test groups, respectively; (B) The proportion of soft tissue samples, vertebral body samples, and pus samples in the positive and negative mNGS test groups (***χ ^2^ test; *P*<0.001). mNGS, metagenomic next-generation sequencing.

Logistic regression identified several factors associated with negative mNGS results. The results of univariate logistic regression are shown in the forest plot (Fig. 2). Needle biopsy was a risk factor [odds ratio (OR): 6.18, 95% confidence interval (CI): 3.33–11.55, *P*<0.01], whereas debridement surgery was protective (OR: 0.16, 95% CI: 0.09–0.3, *P*<0.01). Vertebral body samples were positively associated with negative results (OR: 3.2, 95% CI: 1.75–5.82, *P*<0.01), while pus samples were protective (OR: 0.42, 95% CI: 0.15–0.95, *P*=0.04). We developed a predictive model demonstrating the clinical benefits of different thresholds, and the results indicated that clinical outcomes could be improved (Supplementary materials, Supplemental Digital Content 1, http://links.lww.com/JS9/D65).

**Figure 2:**
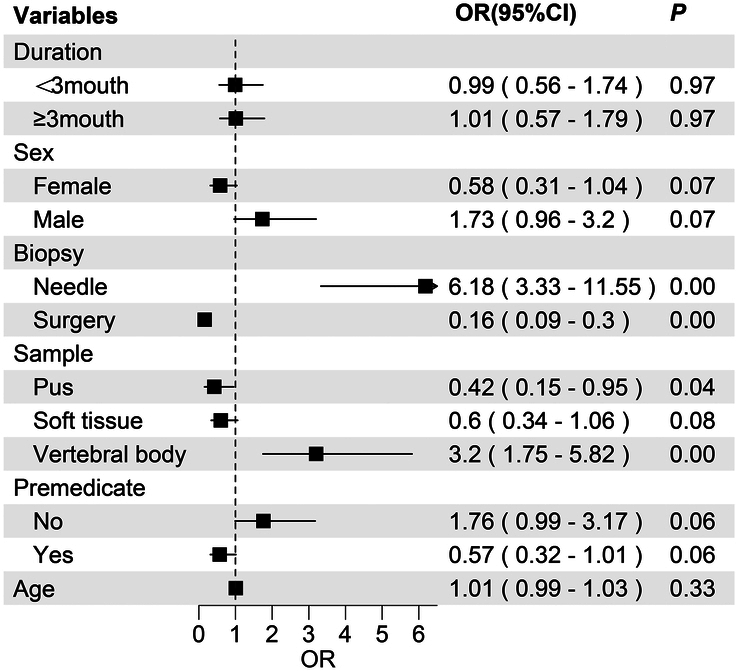
Univariate logistic regression analysis of related factors influencing negative mNGS test results in forest map patients. mNGS, metagenomic next-generation sequencing.

A standardized process for sampling spinal infection lesions has not yet been established, and the effect of the spinal lesion sampling method and sample type on mNGS negative rates has not been reported. Some studies have found that the sampling method and sample type affect the microbial culture positivity rate, but the results are contradictory^7,8^.

There are some areas for improvement in this study, and prospective, multicenter, large-sample clinical studies are needed to validate our results further.

This study is the first to systematically analyze the effect of the surgical sampling method and sample type on the negative rate of mNGS. The results of the study will likely provide a theoretical basis and evidence-based medical evidence for the development of a standardized process for sampling spinal infection lesions.

## Ethical approval

This study was approved by the Ethics Committee of Xiangya Hospital, Central South University (Ethics number: 202212775).

## Consent

Written informed consent was obtained from the patient for publication of this case report and accompanying images. A copy of the written consent is available for review by the editor-in-chief of this journal on request.

## Source of funding

The study was supported by the National Natural Science Foundation of China (No. 82072460) and; Natural Science Foundation of Hunan Province (No. 2023JJ30878).

## Author contribution

G.Z. is responsible for the data curation, visualization, and writing – original draft. Q.L., D.H., X.H. are responsible for collecting clinical data and samples. Y.L., M.T., S.L., and W.L. finished data analysis, investigation, and supervision and provided resources and software. H.Z. and Q.G. supplied the conceptualization, methodology, funding acquisition, resources, supervision, and writing – review and editing. The final version was approved by all authors.

## Conflicts of interest disclosure

Weiqiong Liu is employed by Hunan Sagene Medical Laboratory Co. Ltd. The remaining authors declare that the research was conducted in the absence of any commercial or financial relationships that could be construed as a potential conflict of interest.

## Research registration unique identifying number (UIN)

ChiCTR2400081336.

## Guarantor

Qile Gao, Guang Zhang.

## Data availability statement

Not applicable.

## Provenance and peer review

Not commissioned, externally peer-reviewed.

## Supplementary Material
