# Supplementary material for: Analysis of the clinical factors affecting the negative rate of metagenomic next-generation sequencing in patients with spinal infection
Source: Int J Surg. 2024 Jul 11;111(1):1458–60. doi: 10.1097/JS9.0000000000001913 (PMC11745692; doi:10.1097/JS9.0000000000001913)
Supplement: Supplementary file 1 [file js9-111-1458-s001.doc]

**1．Clinical Data and Sample Characteristics**

We included 340 patients with spinal infections (192 males, 148 females; average age 59.00 [51.00, 68.00] years). Of these, 159 patients had a disease duration of less than 3 months, and 181 had a duration of 3 months or longer. Antibiotics were used by 168 patients before surgery, while 172 did not receive antibiotics. Samples included 275 (80.9%) from debridement surgery and 65 (19.1%) from needle biopsies (Figure 1A), comprising 194 soft tissue samples (57.1%), 79 vertebral tissue samples (23.2%), and 67 pus samples (19.7%) (Figure 1B).


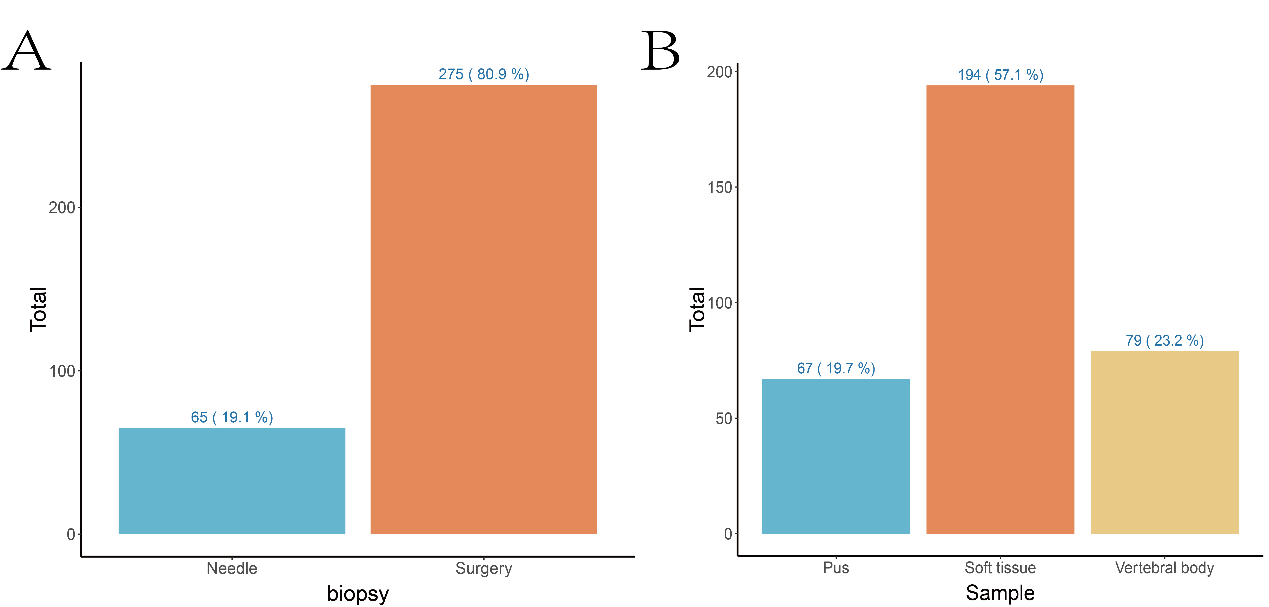


Figure 1 Sample method and sample type bar chart. (A) The bar chart shows the number and proportion of samples obtained by biopsy and debridement respectively; (B) Bar graphs represent the number and overall proportion of soft tissue samples, vertebral samples, and pus samples.

**2. Development and Validation of a Nomogram Model**

Univariate logistic regression identified sampling methods and sample types as factors influencing negative mNGS results. Therefore, we developed a nomogram model to predict negative mNGS results in spinal infection patients, facilitating early clinical decision-making (Figure 2A). The model’s C-index was validated with 10-fold cross-validation (C-index: 0.742, 95% *CI*: 0.510-0.974). Internal validation using an ROC curve showed an AUC of 0.738 (95% *CI*: 0.668-0.809) (Figure 2B). Calibration curves indicated good agreement between predicted and observed outcomes (Figure 2C), with the Hosmer-Lemeshow test confirming good calibration (*X^2^*=1.2625, *P*=0.5319). Decision curve analysis demonstrated the model’s clinical utility across various threshold values (Figure 2D).


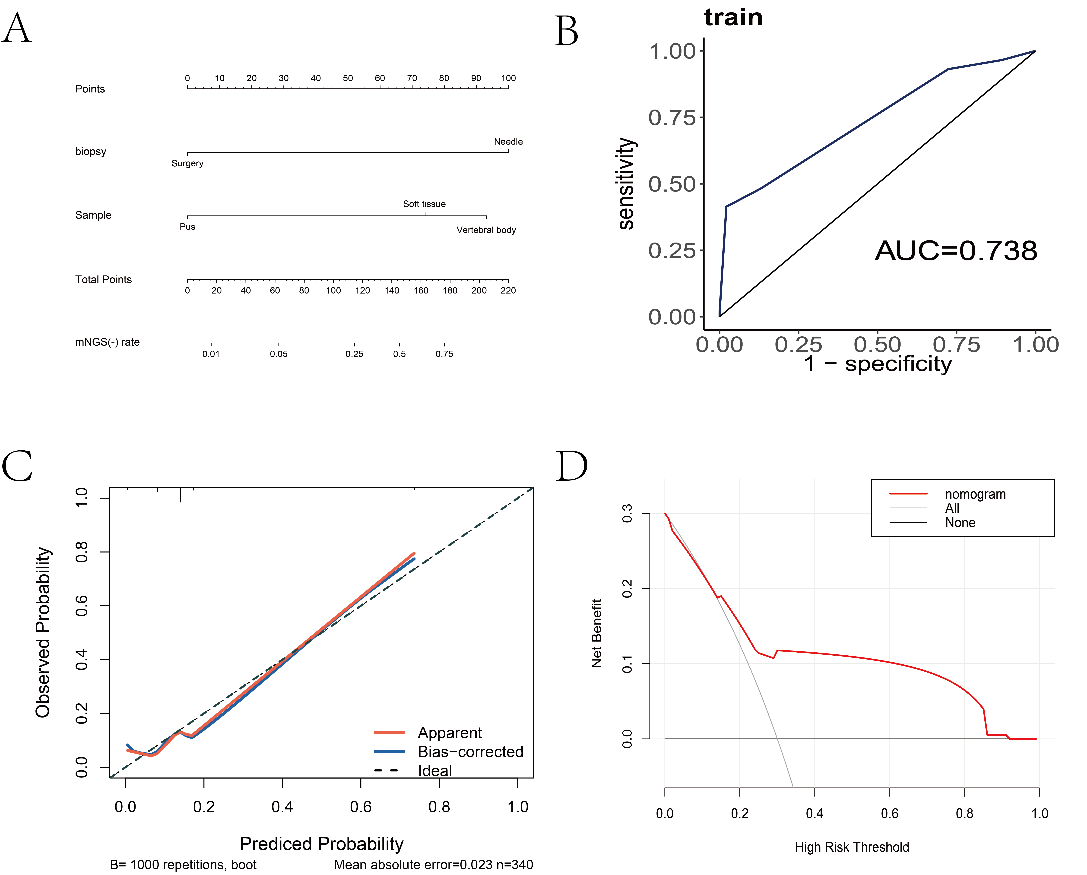


Figure 2 Establishment and verification of negative mNGS prediction model in patients with spinal infection: A. B. ROC curve of the model; C. Calibration curve of the model; D. Decision curve of the model.
